# Effects of parenteral soybean oil lipid emulsion on the long-chain polyunsaturated fatty acid profile in very-low-birth-weight infants

**DOI:** 10.1111/j.1651-2227.2011.02183.x

**Published:** 2011-07

**Authors:** Hiromichi Shoji, Ken Hisata, Mitsuyoshi Suzuki, Naomi Yoshikawa, Hiroki Suganuma, Natsuki Ohkawa, Toshiaki Shimizu

**Affiliations:** Department of Pediatrics, Juntendo University Faculty of MedicineTokyo, Japan

**Keywords:** Arachidonic acid, Docosahexaenoic acid, Fat emulsions, Soybean oil, Very-low-birth-weight infants

## Abstract

**Aim:**

Conventional soybean lipid emulsions contain no docosahexaenoic acid (DHA) or arachidonic acid (AA). We investigated the relationship between blood DHA and AA status in 27 very-low-birth-weight (VLBW) infants with or without parenteral lipid emulsion.

**Methods:**

Sixteen infants received parenteral lipid emulsion, and 11 infants were control group. The fatty acid composition of the erythrocyte membrane was analysed at birth and at 2 weeks of age.

**Results:**

No significant difference in AA levels was observed in the lipid emulsion group between the two time points, whereas the AA levels at 2 weeks were significantly lower than at birth in the control group. The DHA levels in both groups at 2 weeks were significantly lower than at birth, but no group differences were observed at both time points.

**Conclusion:**

The use of parenteral soybean oil lipid emulsions in VLBW infants in the postnatal period may prevent the decline in the AA level but does not appear to influence the DHA level.

## Introduction

Preterm infants, especially very-low-birth-weight (VLBW) infants, often require parenteral lipid emulsion if an adequate energy and lipid intake cannot be achieved by enteral feeding because their polyunsaturated fatty acids (PUFAs) body stores are very low, whereas their metabolic requirements are high. Therefore, a PUFA supply is of critical importance (1).

The conventional lipid emulsion for preterm infants is prepared from long-chain triacylglycerols, mostly soybean oil. The adequacy of soybean lipid emulsions has been questioned, primarily in view of the very high concentrations of linoleic acids (LA; 18:2n-6) and α-linolenic acids (ALA; 18:3n-3) in most commercial preparations. Both of these PUFAs are metabolized to long-chain PUFA (LCPUFA) by desaturation and adding extra double bonds to the carboxyl group end of the molecule.

Docosahexaenoic acid (DHA; 22:6n-3) and arachidonic acid (AA; 20:4n-6) are the major LCPUFA components of membrane lipids in the brain and retina (2), making them essential components of infant nutrition. About 80% of intrauterine DHA and AA accumulation occurs during the last 3 months of pregnancy (2). In preterm infants, the intrauterine supply of DHA and AA is limited by the early termination of maternal-to-foetal fatty acid transfer.

Preterm infants are capable of the *de novo* synthesis of DHA and AA from the dietary essential precursor fatty acids LA and ALA (3,4). However, it is unclear whether the *de novo* synthesis is sufficient to meet their physiological requirements. Failure to accumulate sufficient DHA may impair neurological development (5,6), and clinical evidence supports a relationship between blood AA levels and infant growth (7,8). Soybean-based lipid emulsions contain no DHA or AA. On the other hand, the rationale for quantifying LCPUFA levels in RBC membranes is the assumption that they reflect tissue (including brain) DHA and AA concentrations (9).

Little is known of the relationship between postnatal blood DHA and AA levels in VLBW infants with or without parenteral lipid emulsion. Therefore, we examined the LCPUFA profile of the erythrocyte membrane during the early postnatal period and assessed the efficacy and safety of lipid emulsion in VLBW infants.

## Materials and Methods

### Study population

An observational study was conducted in the NICU of Juntendo University Hospital, Tokyo, Japan. Twenty-seven preterm infants with birth weights <1500 g were enrolled in this study. All infants were born between July 2004 and November 2005. The infants did not have major congenital malformations or metabolic disorders. Gestational age was estimated from the mother’s last menstrual period and confirmed through foetal ultrasound measurements.

**Key notes**

- Soybean lipid emulsion contains no DHA or AA.
- It prevents the decline AA level but does not influence DHA level in VLBW infants.

According to our nutritional protocol, feeding was typically initiated within the first 8 h after birth (20 mL/kg divided over eight feedings per day), and the type of feeding depended on the mother’s willingness or ability to provide breast milk. When breast milk was unavailable, infants received a cow’s milk-based premature formula (Soft-Curd LW®; Meiji Milk Products, Tokyo, Japan) containing LCPUFA. Parenteral nutrition (PN) with dextrose, amino acids (Preamin-P; Fuso Pharmaceutical Industries Ltd, Osaka, Japan) and vitamins was started at birth in all infants. Parenteral lipid emulsion was started from 72 h of age if the infant could not tolerate a sufficient amount (under 50% of total water intake) of feeding.

The infants were divided retrospectively into two groups according to the use of a parenteral lipid infusion. The lipid emulsion group (16 infants; 10 boys and six girls) received the parenteral lipid infusion, and the control group (11 infants; five boys and six girls) did not receive parenteral lipid emulsion because all infants received sufficient (over 50% of total water intake) of feeding before 5 days after birth.

In the lipid emulsion group, the administration of 0.60 ± 0.17 g/kg per day (Intralipos 20%; Ohtsuka Pharmaceutical, Tokyo, Japan) was started from 3 to 8 days (mean 5.1 ± 2.3 days) after birth and continued until the intake of enteral feeding was over 50% of the total water intake for 4 to 18 days (mean 6.9 ± 3.6 days) with the emulsion level being increased to 2.0 g/kg. The composition of the soybean oil lipid emulsion is shown in Table 1.

**Table 1:** Composition of the soybean oil–lipid emulsion (Intralipos® 20%) and infant formula (Soft-Curd LW®).

Intralipos® 20%		
Purified soybean oil	20 mL/100 mL	
Egg phospholipids	1.2 g/100 mL	
Glycerol (anhydrous)	2.2 g/100 mL	
Fatty acid composition (%)	Linoleic acid (18:2n-6)	53
	α-Linolenic acid (18:3n-3)	7
	Oleic acid (18:1n-9)	24
	Palmitic acid (saturated fatty acid)	12
	Stearic acid (saturated fatty acid)	4
Soft-Curd LW®		

Fatty acid composition (mg/100 mL)	Linoleic acid (18:2n-6)	0.45
	α-Linolenic acid (18:3n-3)	0.045
	Arachidonic acid (20:4n-6)	3
	Docosahexaenoic acid (DHA; 22:6n-3)	9.8

All mothers had similar educational backgrounds and were between 25 and 38 years of age. The characteristics of the sampled mothers are presented in Table 2. All were nonvegetarian and had similar dietary habits for the duration of the study. No changes in the dietary habits of the mothers were observed during the study. This study was approved by the Institutional Review Board of Juntendo University Hospital, and written informed consent was obtained from all mothers before inclusion in the study.

**Table 2:** General characteristics of pregnant women and offspring in the lipid emulsion and control groups (mean ± SD)

	Lipid emulsion (n = 16)	Control (n = 11)
Pregnant women		
Age (years)	30.56 ± 4.02	32.45 ± 4.23
Body weight at delivery (kg)	58.62 ± 6.37	55.39 ± 7.97
Body height (cm)	159.56 ± 7.71	157.27 ± 5.37
Weight of placenta (g)	300.15 ± 82.23	308.89 ± 70.66
Offspring		
Sex (M/F)	10/6	5/6
Gestational age (week)	28.87 ± 2.22	30.10 ± 2.32
Birth weight (kg)	0.94 ± 0.26	1.13 ± 0.24*
Body length (cm)	34.63 ± 3.29	36.74 ± 2.77*
Head circumference (cm)	25.62 ± 2.49	26.46 ± 1.76
Apgar score at 5 min	7.87 ± 1.55	8.82 ± 0.75

* p < 0.05 versus the lipid emulsion group.

### Blood sample collection and analysis

Venous blood samples (approximately 600 μL) were collected in EDTA-containing tubes at birth and 2 weeks of age. The samples were centrifuged at 3000 *g* for 10 min. The erythrocyte pellet was washed twice with 0.9% NaCl and stored at −80°C until analysis.

The following biochemical blood variables were assessed at birth and 2 weeks of age by routine laboratory techniques: aminotransferase (AST), alanine aminotransferase (ALT), total bilirubin (T-bil), direct bilirubin (D-bil) and C-reactive protein (CRP).

### Fatty acid composition of the erythrocyte membrane

Erythrocyte lipids were extracted using chloroform and 2-propanol (7:11), according to the method described by Rose and Oklander (10). Erythrocyte fatty acids were esterified by boron trifluoride–methanol. Methyl esters were identified using gas chromatography (Shimadzu GC-17A; Kyoto Japan). The composition of each fatty acid was expressed as the percentage of total fatty acids.

### Statistical analyses

The results are presented as the mean ± standard deviation (SD). Differences between groups were analysed using the Mann–Whitney *U*-test. Differences between the two time points within groups were analysed using paired *t*-tests. p Value less than 0.05 was considered statistically significant. All statistical analyses were performed using StatView 5.0 software (Abacus Concepts, Berkeley, CA, USA).

## Results

Samples from 27 VLBWIs were analysed, and the patient characteristics are summarized in Table 2. The mean birth weight and birth length of the lipid emulsion group were significantly lower than those of the control group. However, no differences in gestational age or head circumference were found between the groups. No significant differences in the time of starting enteral nutrition or the percentage of human milk at 2 weeks were found between the groups. A significant difference in the age of full feeding and the period of parenteral nutrition was observed between the groups (Table 3).

**Table 3:** Nutritional management during the postnatal period in the lipid emulsion and control groups (mean ± SD)

	Lipid emulsion (n = 16)	Control (n = 11)
Initiation of enteral nutrition (h)	10.63 ± 5.49	9.27 ± 6.69
Age at feeds >100 mL/kg per day (days)	11.69 ± 4.75	7.55 ± 3.77*
Period of parenteral nutrition (days)	12.75 ± 5.84	8.91 ± 3.70*
Human milk/enteral nutrition at 2 weeks (%)	91.29 ± 21.55	73.84 ± 39.72
Lipid emulsion		
Initiation (day)	5.13 ± 2.31	–
Amount of initiation (g/kg per day)	0.60 ± 0.17	–
Period (days)	6.88 ± 3.61	–
Total amount of infusion (g/kg)	3.89 ± 3.22	–

* p < 0.05 versus the lipid emulsion group.

**Figure 1:**
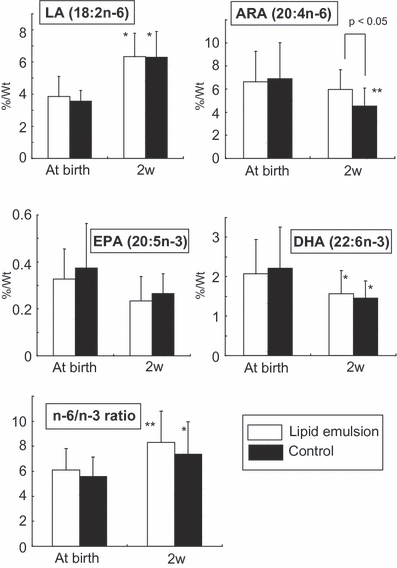
Changes in the fatty acid composition of erythrocytes during the early postnatal period. *p < 0.05; **p < 0.01 compared to at birth.

At 2 weeks of age, the LA levels were significantly higher in both groups than at birth. No significant differences were observed between the groups at the two time points. The ARA levels in the lipid emulsion group remained unchanged throughout the study, whereas the ARA level in the control group was significantly lower at 2 weeks of age compared to at birth. Furthermore, the AA levels in the control group at 2 weeks were significantly lower than in the lipid emulsion group. No significant differences were found in the eicosapentaenoic acid (EPA; 20:5n-3) levels between the time points or groups. At 2 weeks of age, the DHA levels were significantly lower in both groups than at birth. No significant differences were observed between the two groups at the two time points. The n-6/n-3 ratio in both groups at 2 weeks was significantly higher than that at birth, but no between-group differences were found at both time points (Fig. 1).

No significant differences were observed between the groups in the incidence of intracranial haemorrhage (IVH), necrotizing enterocolitis (NEC), period of ventilation, or the levels of transaminases, bilirubin and C-reactive protein at 2 weeks (Table 4).

**Table 4:** Clinical characteristics and serum markers of liver dysfunction, jaundice and sepsis at 2 weeks in the lipid emulsion and control groups

	Lipid emulsion (n = 16)	Control (n = 11)
Antenatal steroids	11	6
Intracranial haemorrhage (III, IV)	0	0
Necrotizing enterocolitis	0	0
Sepsis during first 2 weeks	1	0
Photocoagulation for retinopathy of prematurity	1	1
Period of ventilation (days)	10.64 ± 21.03	10.00 ± 17.18
Oxygen supply at CA 36 weeks of post-conceptional age	3	3
At 2 weeks		
Aminotransferase (IU/L)	19.8 ± 7.27	21.45 ± 6.58
Alanine aminotransferase (IU/L)	8.33 ± 3.09	7.91 ± 2.43
Total bilirubin (mg/dL)	5.32 ± 2.62	6.33 ± 2.40
Direct-bilirubin (mg/dL)	0.84 ± 0.25	1.10 ± 0.46
C-reactive protein (mg/dL)	0.06 ± 0.06	0.04 ± 0.05

## Discussion

Very-low-birth-weight infants are usually treated routinely with total PN containing dextrose, amino acids, lipid emulsions, vitamins and minerals to maintain their nutritional status with the aim of matching normal intrauterine growth during the first few weeks of life.

Lipids are necessary in neonates receiving PN because of their high caloric value, low osmolarity and essential fatty acid (EFA) content (11). However, the endogenous synthesis of LCPUFA from precursor EFA is limited in preterm infants (12), and the large amounts of LA and ALA in soybean oil emulsions may further impair LCPUFA formation via substrate inhibition (13,14).

Simmer and Rao (15) reviewed studies investigating the safety and efficacy of the early introduction of lipid emulsions (within the first 5 days after birth) to parenterally fed premature infants. They found no significant differences between the ‘early’ and ‘no early groups’ for the primary (growth, death and chronic lung disease) and secondary [incidence of respiratory morbidity, NEC, retinopathy of prematurity (ROP), sepsis, IVH and jaundice] outcomes. They concluded that the early introduction of lipids to parenteral nutrition cannot be recommended for short-term growth or to prevent morbidity and mortality in preterm infants. Nevertheless, because a theoretical benefit of the early administration of lipid emulsions exists, early introduction may be preferable.

However, concerns have been raised about the early administration of lipid emulsions because of potential adverse effects including chronic lung disease, increases in pulmonary vascular resistance, impaired pulmonary gas exchange, bilirubin toxicity, sepsis and free radical stress (16). Lipid emulsions contain different amounts of PUFAs, which can act as substrates for the formation of lipid hydroperoxides, mediated by free radicals. The deleterious effects of lipid emulsions on the hepatobiliary system have been suggested recently (11). The accumulation of exogenous lipids in the liver Kupffer cells impairs the clearance of endotoxins, and the peroxidation of lipids produces toxic metabolites (11). Phytosterols contained in lipids emulsions may also have a deleterious effect on biliary secretions (17). Shin et al. (18) also demonstrated that the cumulative amount of lipid infusion was the only significant independent risk factor of PN-associated cholestasis in preterm infants.

In this study, we evaluated the incidence of NEC, ROP, respiratory morbidity, and markers of liver dysfunction, jaundice and sepsis. We did not see any deleterious effect of early lipid emulsion. Furthermore, we previously compared the oxidative stress levels in 23 premature infants by measuring urinary 8-hydroxdeoxyguanosine (8-OHdG; a marker of oxidative DNA damage) and found no significant differences in the urinary 8-OHdG excretion levels between the lipid-infused and not-infused groups at 2, 7 and 14 days of age (19).

As with previous studies (20,21), we found that the levels of DHA and n-3 PUFA during the first 2 weeks decreased regardless of whether soybean oil lipid emulsion was administered. Although preterm infants have limited capacity to convert ALA to DHA, they generally cannot synthesize enough to prevent declines in cellular DHA concentrations without additional supplementation. A newer lipid emulsion (Omegaven) is made from fish oil, which contains LCPUFA (e.g. DHA and EPA). In one study of infants with short bowel syndrome, the reversal of cholestasis because of prolonged PN exposure was quicker in 18 infants who received Omegaven compared to 21 historical controls who received soybean emulsions (9.4 versus 44 weeks) (22). Although randomized controlled trials are needed to determine whether this formulation is more beneficial than the soybean-based lipid emulsion, it offers new possibilities for the management of preterm infants with feeding intolerance. However, this formulation is not available for routine use in Japan.

Our results indicate that parenteral lipid emulsion first administered to VLBW infants around 5 days after birth prevented the decline in the AA level but did not influence the DHA level or n-6/n-3 ratio during the early postnatal period or the short-term clinical outcome. Therefore, we conclude that under the conditions studied, the administration of lipid emulsion to VLBW infants appears safe. Further research is needed to evaluate the long-term outcomes on growth, respiratory morbidity and neurodevelopment.
